# AN ADAPTIVE EQUILIBRIUM REGULATION MODEL OF RESILIENCE

**DOI:** 10.1093/geroni/igz038.1381

**Published:** 2019-11-08

**Authors:** Steve Boker, Steve Boker, Cindy Bergeman

**Affiliations:** 1 University of Virginia, Charlottesville, Virginia, United States; 2 University of Notre Dame, Notre Dame, Indiana, United States

## Abstract

Adaptive equilibrium regulation (AER) models distinguish between the effects of acute versus chronic stressors as a system responds to changes in the environment. Acute stressors have a short time interval during which the stressor is present. Chronic stressors have an onset and may also have an offset, but the stress persists over a period of weeks, months or years. Resilience to an acute stressors may involve rapid self-regulation back to equilibrium without affecting the regulation process itself. Resilience to a chronic stressor may require the system to readapt itself so that regulation of the chronic stressor becomes more effective over time. We present a differential equation model that allows for adaptation of regulation in response to chronic stress and illustrate its use in intensive longitudinal burst data from the Notre Dame Study of Health and Wellbeing.

